# Placental lesions and differential expression of pro-and anti-angiogenic growth mediators and oxidative DNA damage marker in placentae of Ghanaian suboptimal and optimal health status pregnant women who later developed preeclampsia

**DOI:** 10.1371/journal.pone.0265717

**Published:** 2022-03-21

**Authors:** Enoch Odame Anto, David Antony Coall, Emmanuel Akomanin Asiamah, Osei-Owusu Afriyie, Otchere Addai-Mensah, Yaw Amo Wiafe, WKBA Owiredu, Christian Obirikorang, Max Efui Annani-Akollor, Nicholas Akinwale Titiloye, Eric Adua, Emmanuel Acheampong, Evans Asamoah Adu, Stephen Opoku, Agartha Odame Anto, Augustine Tawiah, Youxin Wang, Wei Wang

**Affiliations:** 1 Department of Medical Diagnostics, College of Health Sciences, Kwame Nkrumah University of Science and Technology, Kumasi, Ghana; 2 School of Medical and Health Sciences, Edith Cowan University, Perth, Western Australia; 3 Centre for Precision Health, ECU Strategic Research Centre, Edith Cowan University, Perth, Australia; 4 Department of Medical Laboratory Sciences, School of Allied Health Sciences, University of Health and Allied Sciences, Ho, Ghana; 5 Department of Pathology, Ghana Police Hospital, Accra, Ghana; 6 Department of Pathology, Komfo Anokye Teaching Hospital, Kumasi, West Africa; 7 Department of Molecular Medicine, Kwame Nkrumah University of Science and Technology, Kumasi, Ghana; 8 Rural Clinical School, Medicine and Health, University of New South Wales, Wagga Wagga, Australia; 9 Department of Nursing and Midwifery, School of Allied Health Sciences, University of Health and Allied Sciences, Ho, Ghana; 10 Department of Obstetrics and Gynaecology, Komfo Anokye Teaching Hospital, Kumasi, West-Africa, Ghana; 11 Beijing Key Laboratory of Clinical Epidemiology, School of Public Health, Capital Medical University, Beijing, China; Colorado State University, UNITED STATES

## Abstract

**Background:**

Angiogenic growth mediators (AGMs) and oxidative stress (OS) both play essential roles in normal placental vascular development and as such, placental alterations in these factors contribute to pre-eclampsia (PE). Suboptimal health status (SHS), an intermediate between health and disease, has been associated with imbalanced AGMs and OS biomarkers. Thus, SHS pregnant women may be at increased risk of developing PE and may present abnormal placental alteration and expression of AGMs and OS compared to optimal health status (OHS) pregnant women. We examined the histopathological morphology, immunohistochemical expression of AGMs antibodies and oxidative DNA damage marker in the placentae of SHS and OHS pregnant women who developed early-onset PE (EO-PE) and late-onset (LO-PE) compared to normotensive pregnancy (NTN-P).

**Methods:**

This nested case-control study recruited 593 singleton normotensive pregnant women at baseline (10–20 weeks gestation) from the Ghanaian Suboptimal Health Status Cohort Study (GHOACS) undertaken at the Komfo Anokye Teaching Hospital, Ghana. Socio-demographic, clinical and obstetrics data were collected, and a validated SHS questionnaire-25 (SHSQ-25) was used in classifying participants into SHS (n = 297) and OHS (n = 296). Participants were followed until the time of PE diagnosis and delivery (32–42 weeks gestation). Blood samples were collected at the two-time points and were assayed for AGMs; soluble fms-like tyrosine kinase-1 (sFlt-1), placental growth factor (PIGF), vascular endothelial growth factor-A (VEGF-A), and soluble endoglin (sEng), and OS biomarkers; 8-hydroxydeoxyguanosine (8-OHdG), 8-epiprostaglandinF2-alpha (8- epi-PGF2α) and total antioxidant capacity (TAC) using ELISA. Placental samples were collected for histopathological and immunohistochemical analysis.

**Results:**

Of the 593 pregnant women, 498 comprising 248 SHS and 250 OHS women returned for delivery and were included in the final analysis. Of the 248 SHS women, 56, 97 and 95 developed EO-PE, LO-PE and NTN-P, respectively, whereas 14, 30 and 206 of the 250 OHS mothers developed EO-PE, LO-PE and NTN-P, respectively. At baseline, SHS_NTN pregnant women had a significant imbalance in AGMs and OS biomarkers compared to OHS_NTN pregnant women (p<0.0001). At the time of PE diagnosis, SHS_NTN-P women who developed EO-PE, LO-PE, and NTN-P had lower serum levels of P1GF, VEGF-A and TAC and correspondingly higher levels of sEng, sFlt-1, 8-epiPGF2α, and 8-OHdG than OHS-NTN-P women who developed EO-PE and LO-PE, NTN-P (*p*<0.0001). A reduced placental size, increased foetal/placental weight ratio, and a significantly higher proportion of fibrinoid necrosis, infarction, villous fibrin, syncytial knots, calcification, chorangiosis, tunica media/vascular wall hypertrophy and chorioamnionitis was associated with the SHS group who developed PE (EO-PE>LO-PE) more than OHS groups who developed PE (EO-PE>LO-PE) when all were compared to NTN-P (*p*<0.0001). The intensity of antibody expression of PIGF and VEGF-A were significantly reduced, whereas Flt-1, Eng and 8-OHdG were significantly increased in placentae from SHS-pregnant women who developed EO-PE>LO-PE more than OHS- pregnant women who developed EO-PE>LO-PE when all were compared to NTN-P (*p*<0.0001).

**Conclusion:**

Increased lesions, oxidative DNA damage, and imbalanced expression between pro-and anti-AGMs are associated more with SHS-embodied PE placentae rather than OHS-embodied PE subtypes, thus potentially allowing differential evaluation of PE.

## Introduction

Pre-eclampsia (PE) is a devastating hypertensive disorder reportedly accounting for 5 to 8% of maternal and foetal-related morbidity and mortality [1]. It is a complex multisystemic condition characterised by high blood pressure (BP) and proteinuria noticeable after 20 weeks of gestation [1]. Early-onset pre-eclampsia, which occurs before 34 weeks gestation, is known to be associated with adverse pregnancy events, unlike late-onset pre-eclampsia. Although the exact aetiology of PE is unknown, the pathology of this condition is postulated as a two-stage disorder theory [2–4]. The first stage involves impaired extravillous trophoblast invasion, inadequate remodelling of the maternal spiral arteries and placental under perfusion [2, 3]. The second stage is characterised by increased inflammatory, thrombotic and metabolic responses occurring that cause endothelial dysfunction and multi-organ damage (such as hypertension, renal impairment, liver disorders, brain damage and foetal anomalies) [2, 3]. However, poor placentation is one of the main factors associated with its development [1]. Poor placentation is usually due to uteroplacental hypoperfusion. Furthermore, it is associated with placental infarction and increased fibrin deposition [5]. Placental hypoxia or ischaemia originating from reduced placental perfusion leads to increased oxidative stress and the release of anti-angiogenic mediators into the maternal circulation [6, 7].

Angiogenic growth mediators (AGMs) and oxidative stress (OS) are known to regulate placental vascular development [6]. Particularly, vascular endothelial growth factor (VEGF-A) and placental growth factor (PIGF) are pro-AGMs that regulate vasculogenesis and angiogenesis; hence interference in their function may affect placental development and result in incomplete placental vascular remodelling and angiogenesis [6, 8]. Moreover, soluble Fms-like tyrosine kinase receptor 1 (sFlt-1), an anti-AGM which is a soluble form of VEGF receptor 1, inhibits the function of both PlGF and VEGF and promotes both vasoconstriction and PE development [8]. Increased production of sFlt-1, along with decreased VEGF-A and PlGF in the placenta and maternal circulation, have been related to the pathogenesis of PE [5, 9].

The release of sFlt-1 is stimulated by increasing reactive oxygen species (ROS) formation originating from a hypoxic and ischaemic placenta [6, 10]. Increased ROS subsequently damages proteins, lipids and the DNA leading to OS [11]. One of the effects of the ROS attack on DNA is oxidative DNA damage [11]. Oxidative DNA damage, evidenced by the increased expression of 8-hydroxy-2-deoxyguanosine (8-OHdG), induces vasoconstriction and an increase in BP [12]. Increased placental production of 8-OHdG has been reported in PE women [12, 13]. Despite a positive association of imbalance in OS and AGMs with maternal morbidities and mortalities that have been reported, the aetiology of PE is still not understood. A previous study has indicated the synergistic roles both imbalanced AGMs and increased OS play in placenta-mediated PE development [6]; however, there is a paucity of data investigating the expression of pro-and-anti-AGMs and 8-OHdG in PE collectively in sub-Saharan Africa. Although PE is mostly noticeable after the 20th week of gestation, there is accumulated evidence of early first trimester imbalance of AGMs in pregnant women who are likely to develop PE. While these markers are not readily available in routine clinical setting, there is an urgent need to identify normotensive pregnant women who may have poor health but are without a diagnosable condition. This presentation is also known as subclinical or suboptimal health before the onset of PE. One of the ways to explore poor health is through a suboptimal health perspective.

Suboptimal Health Status (SHS) is the overall physical state between health and disease [14, 15]. SHS explains the subclinical reversible stage of chronic illness and is also characterised by health complaints, general weakness, and low energy within three months [14–16]. Several validated questionnaires are in use for the assessment of SHS; however, the suboptimal health status questionnaire-25 (SHSQ-25), which encompasses five domains, namely: fatigue, cardiovascular health, digestive health, immune health and mental health, are known to be associated with endothelial dysfunction [17], cardiovascular disorders [17, 18] and adverse pregnancy complication such as pre-eclampsia [19]. A Ghanaian cohort study showed that levels of OS biomarkers and AGMs were out of balance among SHS normotensive pregnant women compared to their optimal health status counterparts [20]. Hence, the SHS concept may help evaluate women at risk of developing PE.

Although SHS has been linked with blood pressure disorders, no study to date has integrated the concept of SHS with the differential expression of AGMs and OS biomarkers in placenta and serum of normotensive pregnant women who go on to develop PE. The present study, for the first time in a Ghanaian population, evaluated the histopathological morphology along with the serum levels and immunohistochemical expression of pro-and anti-AGMs and the OS biomarkers in SHS and OHS normotensive pregnant women, who later developed PE, particularly early-onset PE (EO-PE) and late-onset PE (LO-PE).

## Materials and methods

### Study participants

This prospective cohort study was a nested case-control study that initially recruited 593 normotensive pregnant women (NTN-PW) at 10–20 weeks gestation (median gestation, 17 weeks). The participants had no history of the clinically diagnosed disease, without visible proteinuria and hypertension in the last three months and were visiting the antenatal clinic at the Komfo Anokye Teaching Hospital (KATH), Kumasi, Ghana, from June 2017 to December 2019. Pregnant women were initially contacted through a letter of introduction and were invited to an interview and clinical and biochemical evaluation via a phone call. Both nulliparous and multiparous pregnant women aged 18 to 45 years with a singleton pregnancy were contacted. Before inclusion in the study, all participants were physically examined and assessed by a qualified consultant obstetrician/gynaecologist. The study excluded those below 18 years, advanced maternal age (>45years) and twin pregnancy. Also, women with a previous history of pre-eclampsia, gestational diabetes and, gestational hypertension. Additionally, pregnant women with obesity, hyperlipidaemia, cancers, smoking, alcoholism, sexually transmitted infections, sickle cell anaemia, cerebrovascular conditions and cardiovascular conditions of any form were also excluded from the study.

### Ethical consideration

Human research ethical approval for the present study was obtained from the Committee on Human Research Publication and Ethics (CHRPE) of the School of Medical Science (SMS), KNUST and Komfo Anokye Teaching Hospital (KATH) (CHRPE/AP/146/17) and the Human Research Ethics Committee of Edith Cowan University (ECU) (17509). The study was conducted following the guidelines of the Helsinki Declaration. Written informed consent either by a signature or a fingerprint was obtained from all participants.

### Baseline data on sociodemographic, obstetric and clinical assessments

Information regarding socio-demographic characteristics such as age, level of education, occupational history, household income as well as obstetric data (including parity, gravidity, gestational age, contraceptive use, family history of hypertension and previous pregnancy complications) were obtained from the antenatal folder and the participant’s record in the hospital database.

Blood pressure (BP) in mmHg was measured by trained personnel and midwives using mercury sphygmomanometers (Accoson, England). With the aid of a stethoscope, the systolic and diastolic blood pressures were determined and recorded for each patient. The procedure was performed twice at a 5 to 10-minute interval for each participant after the first measurement. The average values of the two measurements were recorded as the BP. Weight and height were read and recorded to the nearest 0.1 kilogram and 0.1 centimetre, respectively. Briefly, pregnant women were made to stand on a weighing scale (Hospibrand ZT-120, England) against the metre rule (Huanan measuring tape) without their shoes, belongings, or extraneous weight. The body mass index (BMI) was calculated with the formula (weight/height2) and written in kg/m^2^ units. The procedure was repeated prior to birth. Early-gestation BMI was recorded from patients’ folders. Proteinuria was measured using a urine reagent dipstick (a semi-quantitative colour scale on the URIT 2VPG Medical electronic Co., Ltd. China). The absence of proteinuria was recorded as ’negative’. The presence of protein in urine was recorded as ≥ 0.3g/l on microalbuminuria or ≥ 1+ on dipstick urinalysis.

### Baseline evaluation of suboptimal health status (SHS)

At baseline [10–20 weeks gestation (average gestation of 17 weeks) or Wave 1 (W1)], the overall health status of 593 NTN-PW was measured using a validated SHSQ-25. The SHSQ-25 comprises five domains: fatigue, cardiovascular health, digestive health, immune health, and mental health. Based on how often each pregnant woman had experienced a particular health complaint in the past three (3) months, they were asked to rate a health statement on a 5-point Likert scale: never or rarely (1), occasionally (2), often (3), very often (4) and always (5).

These scores were recoded as 0–4, followed by a summation of the codes for the 25 answered items. The median of the total score was recorded as the cut-off point, and values ≥ the cut-off represented ’SHS’ (Poor health) and those < the cut-off indicates ’optimal health’ [16]. A median score ≥ 19 depicted SHS in the present study, and <19 depicted optimal health status (OHS). Of the 593 NTN-PW, 297 were SHS, whereas 296 had OHS. A Cronbach’s alpha coefficient value of 0.95 was found after testing the reliability of SHSQ-25.

### Follow-up and the events of pre-eclampsia

**Fig 1:**
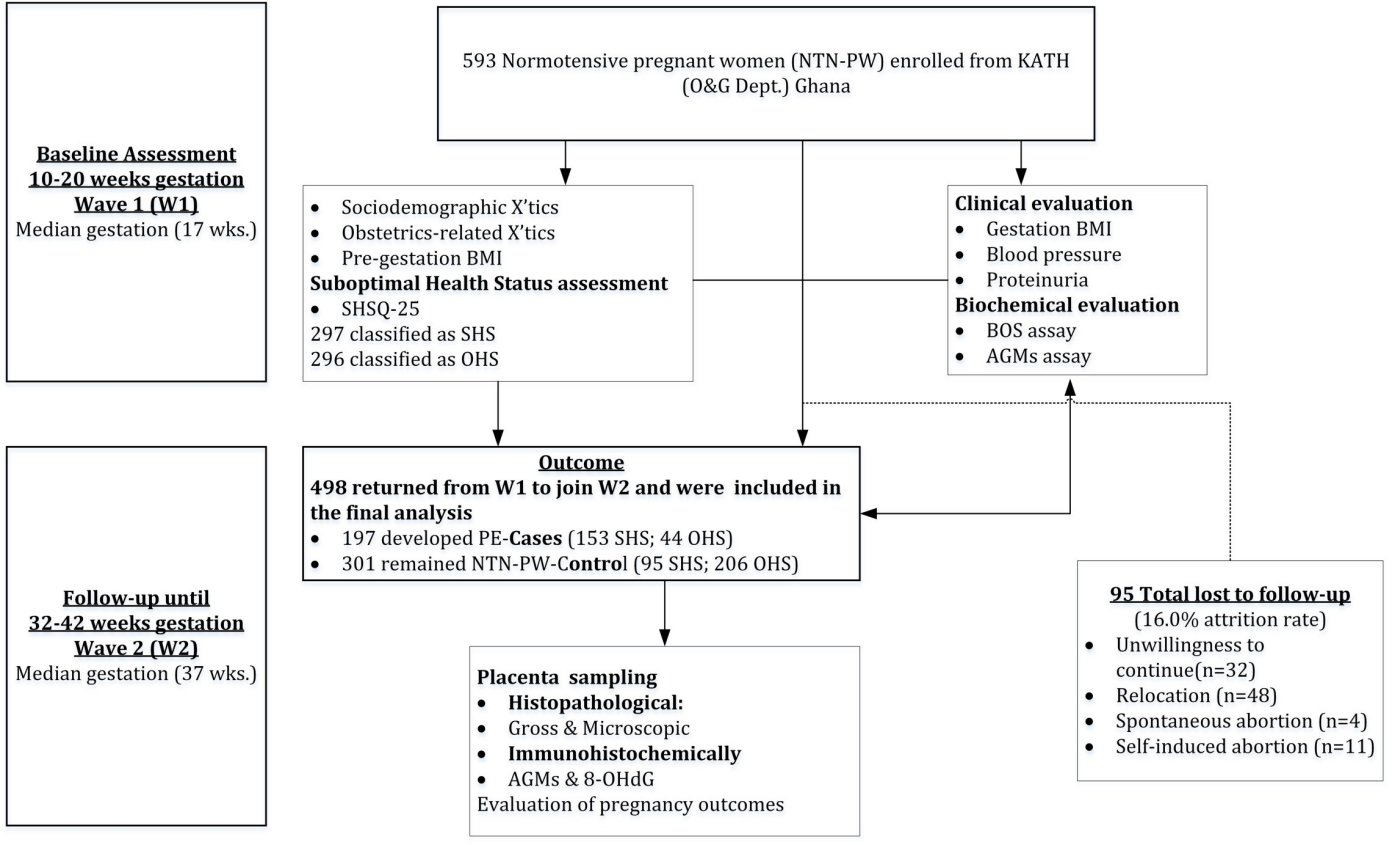
Flowchart of study participants.

The 593 participants (297 SHS and 296 OHS) from baseline (10–20 weeks gestation) were followed until delivery [(32–42 weeks gestation, median gestation 37 weeks, Wave 2 (W2)]. Of the 593 participants, 498 (248 SHS and 250 OHS) returned for delivery and were included in the final assessment. Of the 248 participants who had ’SHS’ at baseline, 153 later developed PE (56 EO-PE and 97 LO-PE), and 95 were NTN-PW. Of the 250 who had ’OHS’ at baseline, 44 developed PE (14 EO-PE and 30 LO-PE), and 206 were NTN-PW. Thus, from the baseline to the time of delivery, 301 of the 593 had normal blood pressure without proteinuria and underlying clinical condition and were classified as control, 197 of the 593 developed PE. These were classified as cases, and 95 were lost to follow-up, resulting in 498 in the final analysis (Fig 1). Reasons for lost-to-follow-up were due to participant’s unwillingness to continue (n = 32), participant relocation (n = 48), self-induced abortion (n = 11) and spontaneous abortion (n = 4). All participants were physically examined and clinically diagnosed by a qualified consultant obstetrician/gynaecologist, followed by laboratory investigations. Pre-eclampsia (PE) diagnosis (ICD-10-CM-014) was based on the presence of proteinuria (≥ 1+ or 0.3g/l) and hypertension (≥ 140/90 mmHg) on two (2) occasions at least four (4) hours apart detected after 20 weeks of gestation in pregnant women who were previously normotensive. Alternatively, PE diagnosis was based on high blood pressure (≥ 140/90 mmHg) combined with the presence of multisystemic manifestations such as HELLP syndrome, renal insufficiency, pulmonary oedema, and visual or cerebral disturbances, which occur even in the absence of proteinuria according to the International Society for the Study of Hypertension in Pregnancy (ISSHP) definition [21]. Pregnant women with PE were classified as early-onset PE (EO-PE) and late-onset PE (LO-PE) when PE diagnosis occurred before and at or after 34 weeks gestation, respectively [21, 22].

### Blood sample collection and OS and AGMs analysis

Fasting venous blood samples were collected in the morning hours (8 am to 11 am) from each pregnant woman up to a total of 2 visits or two waves, at 10–20 weeks gestation and 32–42 weeks gestation into vacutainer® tubes. Serum was separated into two cryovials and stored at -80°C (Thermo Scientific™ Freezers, USA) until the biomarkers of OS and AGMs were analysed.

Following the manufacturer’s instructions, urinary and serum 8-hydroxydeoxyguanosine (8-OHdG) were analysed in duplicate using highly sensitive and competitive ELISA kits (ab201734, Abcam, China). Serum concentrations of 8-OHdG were measured immediately after sample collection to avoid autoxidation during prolonged storage. The inter-and-intra assay coefficients of variation (CV) were 3.5% and 4.5%, respectively. Urinary 8-OHdG concentrations were normalised to creatinine (Cr) concentrations and recorded as ng/mg Cr. Serum 8-epiprostaglandinF2-alpha (8- epi-PGF2α) was analysed in duplicate using competitive ELISA kits from ELabscience, China (cat. log E-EL-0041). The intra-and-inter assay coefficients of variation (CV) were 5.6% and 6.4%, respectively.

Total antioxidant capacity (TAC) reagents were obtained from Sigma-Aldrich (Hong Kong, China). Plasma samples were thawed to measure TAC spectrophotometrically at 593 nm using Mindray BA-88A, China. The estimation of TAC was based on the Ferric Reducing Ability of Plasma (FRAP) and the protocol as described by Benzie and Strain (1996). The absorbance was used to obtain the concentrations after comparison to standard curves and recorded in μmol/l.

AGMs, including serum concentrations of soluble fms-like tyrosine kinase-1 (sFlt-1), placental growth factor (PIGF), vascular endothelial growth factor-A (VEGF-A), and soluble endoglin (sEng), were measured in duplicates using competitive Quantikine ELISA kits (R&D Systems Inc. Minneapolis, MN USA). Absorbance was measured at 450nm wavelength using a microplate ELISA reader (Bio-Tek ELx808 microplate reader, Hayward, CA, USA). The inter-and-intra assay coefficient of variation obtained in our laboratory was 1.1 and 1.3 for VEGF-A, 1.5 and 3.8 for sFlt-1, 4.6 and 3.3 for PlGF and 2.8 and 5.2 for sEng, respectively.

All laboratory assays were performed at the Molecular Medicine Laboratory of the Kwame Nkrumah University of Science and Technology and the Biochemistry and Immunology Department of the Komfo Anokye Teaching Hospital, Ghana.

### Placental tissue collection and histopathological evaluation

Placentae of participants were collected immediately after delivery from the operating theatre and labour wards. The placentae were rinsed in sterile phosphate-buffered saline (PBS), drained, and dried with blotting paper before weighing them. A randomised sampling technique was used to select two tissue sections (full thickness) each from areas such as placental villi, intervillous space, umbilical cord (maternal and foetal ends), and chorioamniotic membrane roll. The tissue segments were then stored in a 10% formalin-saline solution for 48 hours and then processed using an automated tissue processor (Microm STP 120, Thermo Fisher Scientific, GmbH).

Histologic sections, 4 micrometres (μm) thick of paraffin-wax embedded tissues, were sectioned on a rotary microtome (RM2235 Leica Microsystems, GmbH), placed onto glass microscope slides (Leica BONDTM Plus) before air drying then staining with Haematoxylin and Eosin (H&E). The placental histological examinations were performed blindly by two consultant pathologists using a light microscope (Olympus BX51, Japan) and x10, x20 and x40 objective lenses. Histopathological evaluation was based on the presence of the following features: massive perivillous fibrin deposition, fibrinoid necrosis, calcifications and the number of syncytial knot formations per low power field. Each feature was given a subjective score from 0 to 4, and a score of 0 represented "not present", 1 "mild", 2 "moderate" and 3–4 "extensive".

### Immunohistochemical (IHC) analysis of placental tissue

Serial sections of paraffin-embedded placental tissues were cut at four (4) μm thick and placed on IHC adhesion slides (SuperFrost PlusTM, Thermo Fisher Scientific, GmbH) left overnight at ambient temperature, followed by deparaffinisation per standard protocol. A citrate-based (pH 6.0) epitope retrieval solution was used for the heat-induced epitope retrieval (HIER) process, after which all the sectioned slides were pre-conditioned by exposing them to endogenous peroxidase blocker (3% H_2_O_2_) solution.

The sections were then stained with rabbit polyclonal anti-8-OHdG, anti-PIGF, anti-VEGF-A, anti-Flt-1 and anti-Eng. Antibody for 8-OHdG (bs-1278R, 1μg/ml, 1/600), PIGF (bs-0280R, 1μg/ml, 1/600), Flt-1(bs-0170R, 1μg/ml, 1/600), and Eng (bs-4609R, 1μg/ml, 1/600) were purchased from BIOSS antibodies whereas VEGF-A (ab51745, 1.5μg/m, 1/200) was purchased from Abcam and evaluated according to their respective dilutions. The expression of oxidative DNA damage biomarker (8-OHdG) and pro-AGMs (PIGF and VEGF-A) and anti-AGMs (Flt-1 and Eng) in the placental tissues were evaluated immunohistochemically according to the manufacturer’s protocol and supported by the method described by Weel et al. [5]. Photomicrographs were taken from ten randomly selected high-power fields devoid of infarct as hotspots in every section of the placenta with a 20x and 40x objective lens (Olympus BX51, Japan). The degree of IHC positivity staining was evaluated via a count of the number of positive cells and defined as diffused (>50 cells), partial (5–49 cells), and focal positivity (1–5 cells). In contrast, negative staining was <1 cell or no staining. The quantification of the expression of the staining intensity was determined as the number of positive cells per high power field and was analysed by employing Image J software and recorded in pixels/μm/area.

All processing and staining analyses were performed at the Department of Pathology, Komfo Anokye Teaching Hospital, Ghana. Imaging analysis was confirmed at the Molecular and Imaging Laboratory of Edith Cowan University, Australia

### Statistical analyses

The normality of the data was tested using the Kolmogorov-Smirnov test. Data were presented as median (interquartile ranges) for non-parametric continuous variables, mean ± SD for parametric continuous variables and frequency (percentages) for categorical variables. A Chi-square test was performed to test associations between categorical variables. A median comparison between two independent variables (SHS and OHS) was performed using the Mann-Whitney U-test, whereas a mean comparison between two independent variables was performed using an independent sample t-test. A median comparison between more than two independent variables (SHS_EO-PE, SHS_LO-PE, SHS_NTN-P, OHS_EO-PE, OHS_LO-PE, and OHS_NTN-P) was performed using the Kruskal-Wallis test, whereas a mean comparison between more than two independent variables was performed using a one-way ANOVA followed by a Bonferroni post-hoc multiple comparison test and the adjusted *p*-values recorded. *P*<0.05 was considered statistically significant. Data were analysed using GraphPad Prism version 8.

## Results

### Baseline characteristics of study participants

**Table 1** shows the baseline (10-20weeks gestation) socio-demographic, obstetrics, clinical and Biomarker characteristics of normotensive pregnant women who completed the study stratified by Suboptimal Health Status (SHS) and Optimal Health Status (OHS). A total of 593 normotensive pregnant women (NTN-PW) were assessed at baseline, of which 297 had suboptimal health status (SHS) whereas 267 had optimal health status (OHS). Of these, 498 comprising 248 SHS (49.8%) and 250 OHS (50.2%) returned and were enrolled in the final analysis. At baseline, a higher proportion of NTN-PW had completed secondary education (41.8%), were in marriage (83.5%), were Akans (81.7%) by ethnicity, had an informal occupation (66.1%), had low basic monthly income (38.4%), were nulliparous (36.9%), were primigravida (46.8%), and had no history of family hypertension (77.9%), spontaneous abortion (72.1%) and caesarean section (80.1%). When the 498 NTN-PW were stratified into SHS and optimal health status (OHS) from baseline, NTN pregnant women with SHS had a significantly increased history of spontaneous abortion (36.7% vs 19.2%; *p* = 0.0001), nulliparity (41.1% vs 32.8%; *p* = 0.0202) and primigravida (62.9% vs 30.8%; *p*<0.0001) compared to those with OHS. None of the NTN pregnant women had proteinuria at baseline. There was a statistically significant difference between the mean level of SBP among NTN pregnant women with SHS compared to those with OHS (116.0 vs 113.2; *p* = 0.0036). Meanwhile, there was no statistically significant difference between the mean age, gestational age, DBP, pre-gestational and gestational BMI among SHS_NTN pregnant women compared to OHS (*p*>0.05). At baseline, SHS_NTN pregnant women had significantly lower levels of P1GF, VEGF-A and TAC but significantly higher levels of sEng, sFlt-1, 8-epiPGF2α, and 8-OHdG compared to OHS-NTN_pregnant women (*p*<0.0001) **(Table 1)**.

**Table 1:** Baseline (10-20weeks gestation) socio-demographic, obstetrics, clinical and biomarker characteristics of normotensive pregnant women who completed the study stratified by Suboptimal Health Status (SHS) and Optimal Health Status (OHS).

	Normotensive pregnant women (NTN-PW)				
Variable	Total (N = 498)	SHS (N = 248)	OHS (N = 250)	Statistics	p-value
**Age (years)**	29.64 ± 5.98	29.42 ± 5.92	29.60 ± 6.08	0.667	0.5049
**Gestational age (weeks)**	16.98 ± 2.01	16.97 ± 2.08	17.04 ± 1.98	0.060	0.9586
**SBP (mmHg)**	114.7 ± 10.57	116.0 ± 11.00	113 .2 ± 10.01	2.703	**0.0036**
**DBP (mmHg)**	72.58 ± 9.26	73.0 ± 8.78	71.8 ± 8.42	1.618	0.1341
**Early-gestational BMI (Kg/m** ^**2**^ **)**	27.04 ± 4.83	26.86 ± 4.74	27.07 ± 4.92	0.405	0.6887
**Gestational BMI (Kg/m2)**	27.33 ± 4.81	27.32 ± 4.74	27.2 ± 4.92	0.298	0.7658
**Marital Status**				0.207, 2	0.9018
Never married	78(15.7)	37(14.9)	41(16.4)		
Married	416(83.5)	209(84.3)	207(82.8)		
De-facto	4(0.8)	2(0.8)	2(0.8)		
**Ethnicity**				2.768, 3	0.4288
Akan	407(81.7)	196(79.0)	211(84.4)		
Ga-Adangbe	9(1.8)	6(2.4)	3(1.2)		
Mole Dagbani	75(15.1)	42(16.9)	33(13.2)		
Ewe	7(1.4)	4(1.6)	3(1.2)		
**Highest Level of Education**				1.794, 3	0.6163
Unschooled	3(0.4)	1(0.4)	2(0.8)		
Primary	168(33.7)	82(33.1)	86(34.4)		
Secondary	208(41.8)	110(44.4)	98(39.2)		
Tertiary	119(23.9)	55(22.2)	64(25.6)		
**Basic income (GH₡)**				2.777, 3	0.4273
None	47(9.4)	28(11.3)	19(7.6)		
Low (<500.0)	191(38.4)	92(37.1)	99(39.6)		
Middle (500.0–1000.0)	170(34.1)	87(35.1)	83(33.2)		
High (>1000.0)	90(18.1)	41(16.5)	49(19.6)		
**Parity**				7.706, 2	**0.0212**
Nulliparous	184(36.9)	102(41.1)	82(32.8)		
Primiparous	135(27.1)	54(21.7)	81(32.4)		
multiparous	179(36.0)	92(37.1)	87(34.8)		
**Gravidity**				51.54, 1	**<0.0001**
Primigravida	233(46.8)	156(62.9)	77(30.8)		
Multigravida	265(53.2)	92(37.1)	173(69.2)		
**FH of Hypertension** (Yes)	110(22.1)	57(23.0)	53(21.2)	0.230,1	0.6314
**History of SA** (Yes)	139(27.9)	91(36.7)	48(19.2)	5.083, 1	**0.0001**
**Previous CS** (Yes)	99(19.9)	48(19.4)	51(20.4)	0.085, 1	0.7701
**No proteinuria (<0.3g/g/24hr)**	498(100.0)	248(100.0)	250(100.0)		1.0000
**Biomarkers of AGMs**					
Serum PlGF (pg/ml)	97.3(80.3–102.9)	90.5(70.4–100.8)	98.6(89.6–105.5)	23521	<0.0001
Serum VEGF-A (pg/ml)	171.5(124.1–191.4)	138.7(106.4–189.1)	185.1(148.7–199.2)	20856	<0.0001
Serum sEng (ng/mL)	4.9(3.4–6.5)	5.2(3.9–7.4)	4.1(3.3–5.8)	23016	<0.0001
Serum sFlt-1 (pg/ml)	611.2(462.9–797.1)	673.8(486.5–906.5)	562.4(446.9–717.5)	23020	<0.0001
**OS biomarkers**					
Serum 8-epiPGF2α (pg/ml)	421.0(318.0–545.1)	466.2(337.3–600.1)	387.5(300.4–496.1)	23151	<0.0001
Serum 8-OHdG (ng/L)	79.9(61.8–84.3)	82.5(70.9–89.1)	74.7(53.6–83.0)	22819	<0.0001
Plasma TAC (μmol/l)	237.5(178.6–305.6)	228.3(176.1–289.9)	246.5(180.0–314.8)	28116	0.0371

Values are presented as mean ± SD, frequency (percentage), SA spontaneous abortion, CS: caesarean section; GH₡: Ghana cedi; SBP: systolic blood pressure; DBP: diastolic blood pressure. OHS: optimal health status.

### Demographic data, clinical and adverse pregnancy outcomes of SHS and OHS- pregnant women who developed (PWD) EO-PE and LO-PE compared to NTN-P

**Table 2** shows the demographic data, clinical and adverse pregnancy outcomes of SHS and OHS-PWD EO-PE and LO-PE compared to NTN-P. Regarding maternal age, the SHS pregnant women who developed EO-PE were older than those who developed LO-PE and significantly different compared to NTN-P (*p*<0.0001). Conversely, there was no statistically significant difference in maternal age among OHS pregnant group (*p* = 0.0731). The median gestational age and birthweight were statistically significantly lower at delivery. In contrast, levels of Systolic BP (SBP) and Diastolic BP (DBP) were significantly higher in SHS rather than OHS pregnant women who developed EO-PE followed by those who developed LO-PE when they were both compared to NTN-PW (*p*<0.0001). A significantly higher proportion of SHS rather than OHS pregnant women who developed EO-PE followed by those who developed LO-PE had a preterm birth, foetal growth restriction (FGR), stillbirth, foetal demise, preterm premature rupture of membrane (PPROM) and APGAR score below seven after 5 minutes when both were compared to NTN-P (*p*<0.0001). Among SHS cohorts, SHS-pregnant women who developed EO-PE rather than SHS- pregnant women who developed LO-PE had significantly lower levels of serum P1GF, VEGF-A and TAC (p< 0.0001) and correspondingly significantly higher levels of sEng, sFlt-1, 8-epiPGF2α and 8-OHdG, when both were compared to SHS- pregnant women who experienced NTN-pregnancy (*p*<0.0001). Among OHS cohorts, OHS-pregnant women who experienced NTN-pregnancy had significantly higher levels of serum P1GF, VEGF-A and TAC and correspondingly significantly lower levels of serum sEng, sFlt-1, 8-epiPGF2α, and 8-OHdG compared to OHS-pregnant women who developed LO-PE and OHS-pregnant women who developed EO-PE (*p*<0.0001). Intergroup analysis indicated a massive imbalance in AGMs and OS biomarkers among the SHS group rather than the OHS group, even though no statistically significant differences were observed except for PIGF levels **(Table 2).**

**Table 2:** Demographic data, clinical and adverse pregnancy outcomes of SHS and OHS-PWD EO-PE and LO-PE compared to NTN-P.

	SHS				OHS			
Characteristics	SHS-PWD EO-PE (N = 56)	SHS-PWD LO-PE (N = 97)	SHS-PWD NTN-P (N = 95)	#*p*-value	OHS-PWD EO-PE (N = 14)	OHS-PWD LO-PE (N = 30)	OHS-PWD NTN-P (N = 206)	#*p*-value
**Maternal age (yrs)**	34(21.8–38.8) * ¥	28.0(24.0–33.0)	30.0(27.0–34.0)	**<0.0001**	29.0(26.0–33.0)	28.5(22–33.5)	30.0(26.0–34.0)	0.0731
**SBP (mmHg)**	180(168–189) * ¥	160(156–180) * ‡	120(114–122.0) †	**<0.0001**	164(160.0–184.0) ǂ	148(159–173) ǂ	116(114.0–121.0)	**<0.0001**
**DBP (mmHg)**	105(100–113.0) *	104(100.0–110.0) *	78(70.0–80.0)	**<0.0001**	103(100.0–113.0) ǂ	101(100–110) ǂ	76.0(69.0–80.0)	**<0.0001**
**GA at delivery**	32(32–33) *	37.0(35.0–38.0) *	38(37.0–39.0)	**<0.0001**	33.0(32.8–33.0) ǂ	36(34–37) ǂ	38.0(37.0–39.0)	**<0.0001**
**Baby birthweight**	1884(1375–2150)*	2133(1500–2800) *‡	2450(2100–3100) †	**<0.0001**	1986(1200–2100) ǂ	2331 (1900–3000) ǂ	2940 (2600–3300)	**<0.0001**
**Preterm delivery**	39(69.6) *	20(20.6) *‡	5(5.3)	**<0.0001**	8(57.1) ǂ	15(50.0) ǂ	8(3.9)	**<0.0001**
**FGR**	33 (58.9) *¥	12 (12.4) *‡	9 (9.5)	**<0.0001**	6 (42.9) ǂ	8 (26.7) ǂ	3 (14.6)	**<0.0001**
**Stillbirth**	11 (19.6) *	8 (8.2) *‡	5 (5.3)	**<0.0001**	2 (14.3) ǂ	1 (3.3) ǂ	2 (0.9)	**<0.0001**
**Foetal demise**	5 (8.9) *¥	1 (1.0) *	3 (3.2)	**<0.0001**	3 (21.4) ǂ	0 (0.0)	0 (0.0)	**<0.0001**
**PPROM**	3 (5.3) *¥	1 (1.0) *	6 (6.3)	**<0.0001**	2 (14.3) ǂ	1(3.3) ǂ	0(0.0)	**<0.0001**
**APGAR≤7 at 5min**	13 (23.2) *	7 (7.2) *‡	1 (1.1)	**<0.0001**	2 (14.3) ǂ	1 (3.3) ǂ	0 (0.0)	**<0.0001**
**Mode of delivery**				**<0.0001**				**<0.0001**
*SVD*	15 (26.8)	73 (75.3)	81 (85.3)		6 (42.9)	25 (83.3)	194 (94.2)	
*Caesarean section*	41 (73.2)	24 (25.8)	16 (16.8)		8 (57.1)	5 (16.7)	12 (5.8)	
**AGMs**								
PlGF (pg/ml)	71.5(45.2–93.3)* ¥	73.8(42.4–95.6)* ‡	104.6(97.5–109.9)†	**<0.0001**	83.3(51.4–96.9)ǂ	90.1(62.2–102.2)ǂ	103.5(96.9–110.2)	**<0.0001**
VEGF-A (pg/ml)	110.8(82.4–172.4)*	114.0(71.2–137.6)*	200.6 (182.0–212.6)	**<0.0001**	118.9(68.2–180.7) ǂ	143.1(93.0–186.2)ǂ	201.8(182.3–230.7)	**<0.0001**
sEng (ng/mL)	11.9(10.5–14.3) *	10.7(8.9–13.5) *	8.8(7.9–9.9)	**<0.0001**	11.3(9.0–13.2)ǂ	10.3(7.7–12.9)ǂ	8.7(7.6–10.2)	**<0.0001**
sFlt-1 (pg/ml)	1290 (898.3–1581) *	1107 (837.8–1389) *	767.5 (623.9–950.0)	**<0.0001**	1142 (796.2–1358) ǂ	885.9 (727.9–1082) ǂ	784.2 (623.8–980.9)	**<0.0001**
**OS biomarkers**								
8-epiPGF2α (pg/ml)	2560(2057–3115)*	2187(1599–2882) *	1466 (1215–1839)	**<0.0001**	2223 (1489–2636) ǂ	1625 (1428–2067) ǂ	1497 (1185–1894)	**<0.0001**
8-OHdG (ng/L)	142.6 (131.6–155.7) *	136.1 (124.0–151.2) *	118.4 (79.2–133.2)	**<0.0001**	136.4 (132.2–151.4) ǂ	132.1 (115.6–141.9) ǂ	118.4 (82.4–132.9)	**<0.0001**
TAC (μmol/l)	131.4 (109.9–192.3) *	180.9 (119.9–249.5) *	351.6 (261.9–475.8)	**<0.0001**	160.9 (112.4–225.2) ǂ	199.6 (118.8–243.4) ǂ	373.8 (268.5–472.9)	**<0.0001**

Values are presented as median (interquartile ranges, 25^th^ -75^th^). n (%): frequency (proportion). SHS: suboptimal health status; OHS: optimal health status; PWD: pregnant women who developed; EO-PE: early-onset PE; LO-PE: late-onset PE; NTN-P: normotensive pregnancy; GA: gestational age. PPROM: preterm premature rupture of membrane; SVD: spontaneous vaginal delivery; FGR: Foetal growth restriction; APGAR: activity, pulse, grimace, appearance, respiration. P-value <0.05 in bold-face indicates a statistically significant difference.

#p-value indicates p-values within group comparison.

*indicates significance compared to SHS-PWD NTN-PW

ǂ indicates significant compared to OHS-PWD NTN-PW

¥ indicates significance between SHS-PWLD EO-PE and OHS-PWLD EO-PE

‡ indicates significance between SHS-PWLD LO-PE and OHS-PWLD LO-PE

† indicates significance between SHS-PWLD NTN-PW and OHS-PWLD NTN.

### Macroscopic (gross) morphology of the placentae among the SHS and OHS-PWD EO-PE and LO-PE compared to NTN-P

**Table 3** show the macroscopic morphology of the placentae among the SHS and OHS-PWD EO-PE and LO-PE compared to NTN-P. There were statistically significantly lower mean placental weights, placental thicknesses and umbilical cord lengths. Conversely, a statistically significantly increased foetal/placental weight ratio was observed in placentae of SHS rather than OHS pregnant women, who developed EO-PE followed by those who developed LO-PE, when both were compared to NTN pregnant women (*p*<0.0001). Higher proportions of calcified and infarcted areas, the irregular architecture of cotyledons, abnormal cord insertions and membrane rupture sites were associated with placentae of SHS rather than OHS pregnant women who developed EO-PE followed by those who developed LO-PE when both were compared to NTN- pregnant women (*p*<0.0001) **(Table 3).**

3

**Table 3:** Gross morphology of the placentae among the SHS and OHS-PWD EO-PE and LO-PE compared to NTN-P.

Macroscopic	SHS-PWD EO-PE (N = 56)	SHS-PWD LO-PE (N = 97)	SHS-PWD NTN-PW (N = 95)	# *p-value*	OHS-PWD EO-PE (N = 14)	OHS-PWD LO-PE (N = 30)	OHS-PWD NTN-PW (N = 206)	# *p-value*
**Placental weight (g)**	318.0 ± 17.0*	409.0 ± 16.9*	427.0 ± 16.7	**<0.0001**	364.0 ± 17.3ǂ	420.0 ± 17.0ǂ	505.0 ± 17.3	**<0.0001**
**Foeto-placental wt. ratio**	6.7 ± 0.1*	6.3 ± 0.2*	5.5 ± 0.2	**<0.0001**	6.4 ± 0.2ǂ	5.62 ± 0.2ǂ	5.2 ± 0.2	**<0.0001**
**Placental thickness**	1.6 ± 0.0*	1.7 ± 0.1*	1.8 ± 0.0	**<0.0001**	1.7 ± 0.0ǂ	1.83 ± 0.0ǂ	2.1 ± 0.0	**<0.0001**
**Umbilical cord length**	43.1 ± 6.1*	46.3 ± 6.4*	50.2 ± 6.2	**<0.0001**	45.1 ± 6.3ǂ	48.73 ± 5.95ǂ	54.7 ± 6.2	**<0.0001**
**Calcifications**				**<0.0001**				**<0.0001**
Absent	37 (66.1)	86 (88.7)	91 (95.9)		9 (64.3)	28 (93.3)	204 (99.0)	
Present	19 (33.9)	11 (11.3)	4 (4.2)		5 (35.7)	2 (6.7)	2 (1.0)	
**Infarcted villi surface**				**<0.0001**				**<0.0001**
Absent	35 (62.5)	88 (90.7)	94 (98.9)		8(57.1)	29 (96.7)	206 (100)	
Present	21 (37.5)	9 (9.2)	1 (1.1)		6 (42.9)	1 (3.3)	0 (0.0)	
**Irregular architecture of cotyledons**				**<0.0001**				**<0.0001**
Absent	46 (82.1)	94 (96.9)	95 (100)		10(71.4)	28 (93.3)	206 (100)	
Present	10 (17.9)	3 (3.1)	0(0.0)		4(28.6)	2 (6.7)	0 (0.0)	
**Cord insertion**				**<0.0001**				**<0.0001**
Central	48 (85.7)	96(99.0)	95 (100)		9 (64.3)	26 (86.7)	206 (100)	
Marginal	8 (14.3)	1 (1.0)	0(0.0)		5 (35.7)	4 (13.3)	0 (0.0)	
**Membrane rupture site**				**<0.0001**				**<0.0001**
Absent	47 (83.9)	95(97.9)	95 (100)		10 (71.4)	28 (93.3)	206 (100)	
Present	9 (16.1)	2 (2.1)	0(0.0)		4(28.6)	2 (6.7)	0 (0.0)	

Values are presented as Mean ± SD and n (%): frequency (proportion). SHS: suboptimal health status; OHS: optimal health status; PWD: pregnant women who developed; EO-PE: early-onset PE; LO-PE: late-onset PE; NTN-P: normotensive pregnancy. P-value <0.05 in bold-face indicates a statistically significant difference.

#p-value indicates p-values within group comparison.

* indicates significance compared to SHS-PWD NTN-P.

ǂ indicates significance compared to OHS-PWD NTN-P

† indicates significance between SHS-PWD NTN-PW and OHS-PWD NTN-P.

### Photomicrographs of placental villi and stromal histopathological morphology

**Fig 2:**
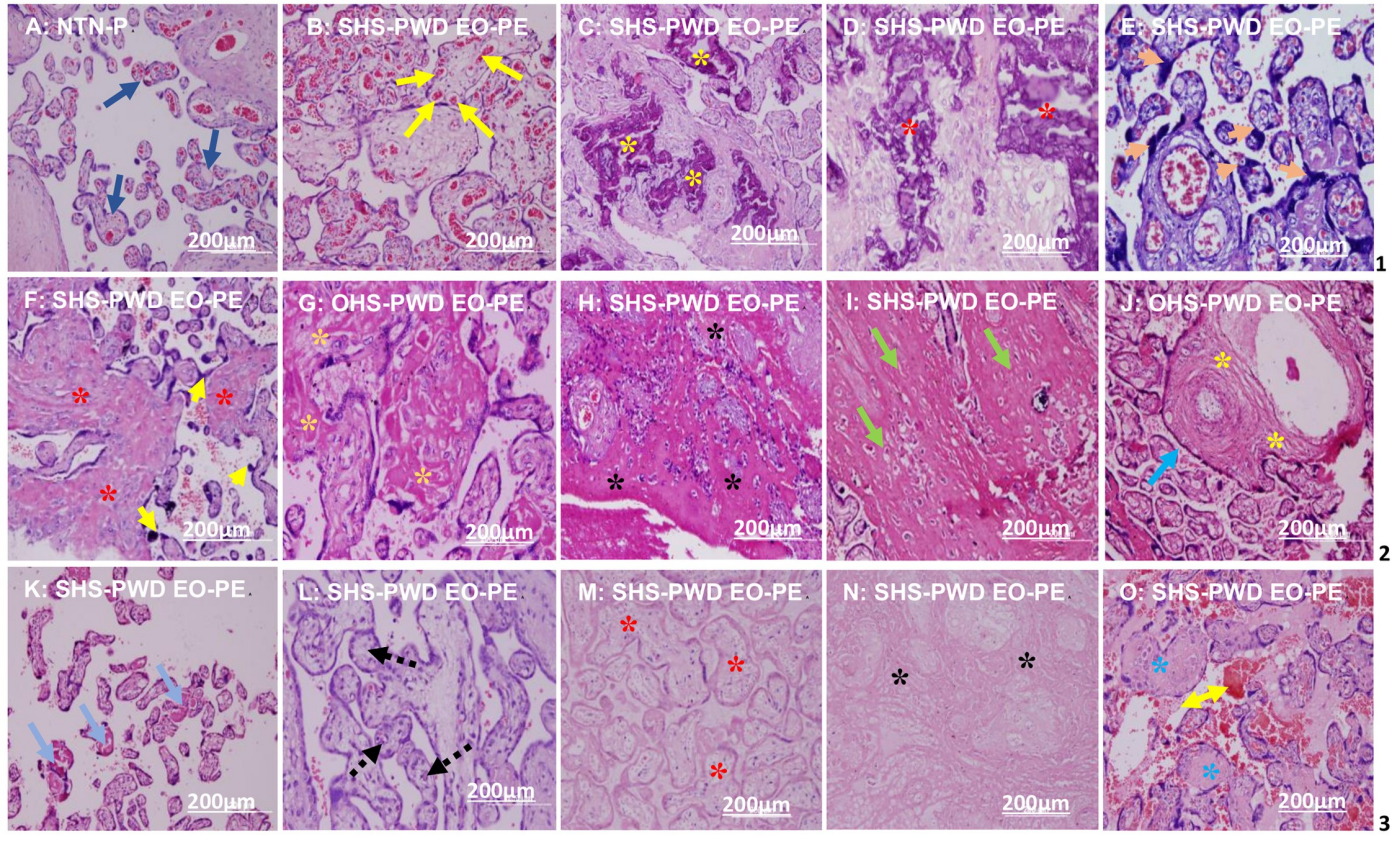
Photomicrographs of placental villi of SHS and OHS-PWD EO-PE and LO-PE compared to NTN-P. A: Normal villi (deep blue arrows, row 1) of NTN-PW at 38 weeks (x20 magnification). B: Extensive chorangiosis (yellow arrows, row 1) in SHS-PWD EO-PE at 32 weeks (x20 magnification). C: massive intervillous (yellow asterisks, row 1) (x20) and D: intravillous calcifications (red asterisks, row 1) (x20 magnification) SHS-PWD EO-PE at 32 weeks. E: massive syncytial knots formation with few fibrins (orange arrowhead) in SHS-PWD EO-PE at 32 weeks (x20 magnification). F: massive intravillous fibrin (red asterisks, row 2) with few syncytial knot formations (yellow arrowhead, row 2) in SHS-PWD EO-PE at 32 weeks (x20 magnification). G: Perivillous fibrin deposition (gold asterisks, row 2) in OHS-PWD EO-PE at 33 weeks (x20). H: extensive perivillous fibrin deposition (black asterisks, row 2) SHS-PWD EO-PE at 32 weeks (x20 magnification). I: extensive extravillous with few areas of infarction (green arrows, row 2) (x20) SHS-PWD EO-PE. J: Large stem villi (sea blue arrow) with extensive fibrin deposition (yellow asterisk, row 2) in OHS-PWD EO-PE at 33 weeks (x20). K: distal villous hypoplasia with few thromboses (blue arrows, row 3) in SHS-PWD EO-PE at 33 weeks (x10 magnification). L: empty capillaries devoid of red blood cells (black line arrows, row 3) in SHS-PWD EO-PE (x20 magnification). M: extensive villous infarction (red asterisk, row 3) in SHS-PWD EO-PE at 32 weeks (x20 magnification). N: extensive fibrinoid necrosis with infarction (black asterisk) in SHS-PWD EO-PE at 32 weeks (x20 magnification). O: Massive intervillous haemorrhage (yellow double arrows) with few avascular villi (sea blue asterisks) in SHS-PWD EO-PE at 32 weeks (x20). Scale bar = 200μm.

**Fig 2** shows the photomicrographs of placental villi of SHS and OHS-PWD EO-PE and LO-PE compared to NTN-P. The histopathological features in the photomicrographs showed moderate to extensive villi and stromal lesions in the placentae of SHS, rather than OHS pregnant women who developed EO-PE. Following were those who developed LO-PE and a mild to no lesion in NTN-pregnant women placentae. The placental lesions commonly identified were villous fibrinoid necrosis, inter and intravillous fibrin deposition, infarction/ hyalinisation, inter-and intravillous calcification, massive syncytial knot formation and chorangiosis (**Fig 2)**.

### Association and histopathological morphology of the placental villi and stroma of SHS and OHS pregnant women who developed EO-PE and LO-PE compared to NTN-P women

As shown in **Table 4,** a statistically significant higher proportion of placental villi and stromal lesions (as depicted in **Fig 2**) were associated with placentae of SHS rather than OHS pregnant women who developed EO-PE followed by those who developed LO-PE when both were compared to NTN-pregnant women placentae (*p*<0.0001) **(Table 4).**

**Table 4:** Histopathological morphology of the placental villi between SHS and OHS-PWD EO-PE and LO-PE compared to NTN-P.

Features	SHS-PWD EO-PE (N = 56)	SHS-PWD LO-PE (N = 97)	SHS-PWD NTN-PW (N = 95)	*#p-value*	OHS-PWD EO-PE (N = 14)	OHS-PWD LO-PE (N = 30)	OHS-PWD NTN-PW (N = 206)	*#p-value*
**Villous fibrinoid necrosis**				**<0.0001**				**<0.0001**
Not present	9(16.1)	76(78.4)	93(97.9)		6(42.9)	22(73.3)	206(100)	
Mild	8(14.3)	14(14.4)	2(2.1)		5(35.7)	8(26.7)	0(0.0)	
Moderate	28(50.0)	4(4.1)	0(0.0)		1(7.1)	0(0.0)	0(0.0)	
Extensive	11(19.6)	3(3.1)	0(0.0)		2(14.3)	0(0.0)	0(0.0)	
**Inter/perivillous fibrin**				**<0.0001**				**<0.0001**
Not present	10(17.9)	71(73.2)	90(94.7)		5(35.7)	18(60.0)	206(100)	
Mild	12(21.4)	10(10.3)	4(4.2)		2(14.3)	10(33.3)	0(0.0)	
Moderate	21(37.5)	7(7.2)	1(1.1)		4(28.6)	1(3.3)	0(0.0)	
Extensive	13(23.2)	5(5.2)	0(0.0)		3(21.4)	1(3.3)	0(0.0)	
**Intravillous fibrin**				**<0.0001**				**<0.0001**
Not present	22(39.3)	83(85.6)	94(98.9)		7(50.0)	24(80.0)	206(100.0)	
Mild	11(19.6)	8(8.2)	1(1.0)		2(14.3)	5(16.7)	0(0.0)	
Moderate	15(26.8)	5(5.2)	0(0.0)		4(28.6)	1(3.3)	0(0.0)	
Extensive	8(14.3)	2(2.1)	0(0.0)		1(7.1)	0(0.0)	0(0.0)	
**Calcification**				**<0.0001**				**<0.0001**
Not present	26(46.4)	88(90.7)	91(95.8)		8(57.1)	26(86.7)	200(97.1)	
Mild	16(28.6)	5(5.2)	3(3.2)		3(21.4)	4(13.3)	6(2.9)	
Moderate	9(16.1)	3(3.1)	0(0.0)		2(14.3)	0(0.0)	0(0.0)	
Extensive	5(8.9)	1(1.0)	0(0.0)		1(7.1)	0(0.0)	0(0.0)	
**Infarction/hyalinised areas**				**<0.0001**				**<0.0001**
Not present	23(41.1)	90(92.8)	94(98.9)		5(35.7)	27(90.0)	206(100)	
Mild	18(32.1)	6(6.2)	1(1.0)		4(28.6)	3(10.0)	0(0.0)	
Moderate	11(19.6)	1(1.0)	0(0.0)		3(21.4)	0(0.0)	0(0.0)	
Extensive	4(7.1)	0(0.0)	0(0.0)		2(14.3)	0(0.0)	0(0.0)	
**Syncytial Knot Formation**				**<0.0001**				**<0.0001**
Not present	25(44.6)	84(86.6)	92(96.8)		6(42.9)	22(73.3)	204(99.0)	
Mild	9(16.1)	5(5.2)	3(3.2)		3(21.4)	6(20.0)	2(0.0)	
Moderate	12(21.4)	7(7.2)	0(0.0)		4(28.6)	2(6.7)	0(0.0)	
Extensive	10(17.9)	1(1.0)	0(0.0)		1(7.1)	0(0.0)	0(0.0)	
**Chorangiosis**				**<0.0001**				**<0.0001**
Not present	41(73.2)	93(95.9)	95(100)		9(64.3)	29(96.7)	206(100)	
Mild	7(12.5)	3(3.1)	0(0.0)		1(7.1)	1(3.3)	0(0.0)	
Moderate	5(8.9)	1(1.0)	0(0.0)		3(21.4)	0(0.0)	0(0.0)	
Extensive	3(5.4)	0(0.0)	0(0.0)		1(7.1)	0(0.0)	0(0.0)	

Values are presented as frequency (percentages). P< 0.05 in bold value indicates statistical significance. Association was tested using Chi-square test for trend. SHS: suboptimal health status; OHS: optimal health status; PWD: pregnant women who developed; EO-PE: early-onset PE; LO-PE: late-onset PE; NTN-PW: normotensive pregnancy women.

### Photomicrographs of umbilical cord and chorioamniotic membrane histopathological morphology

**Fig 3:**
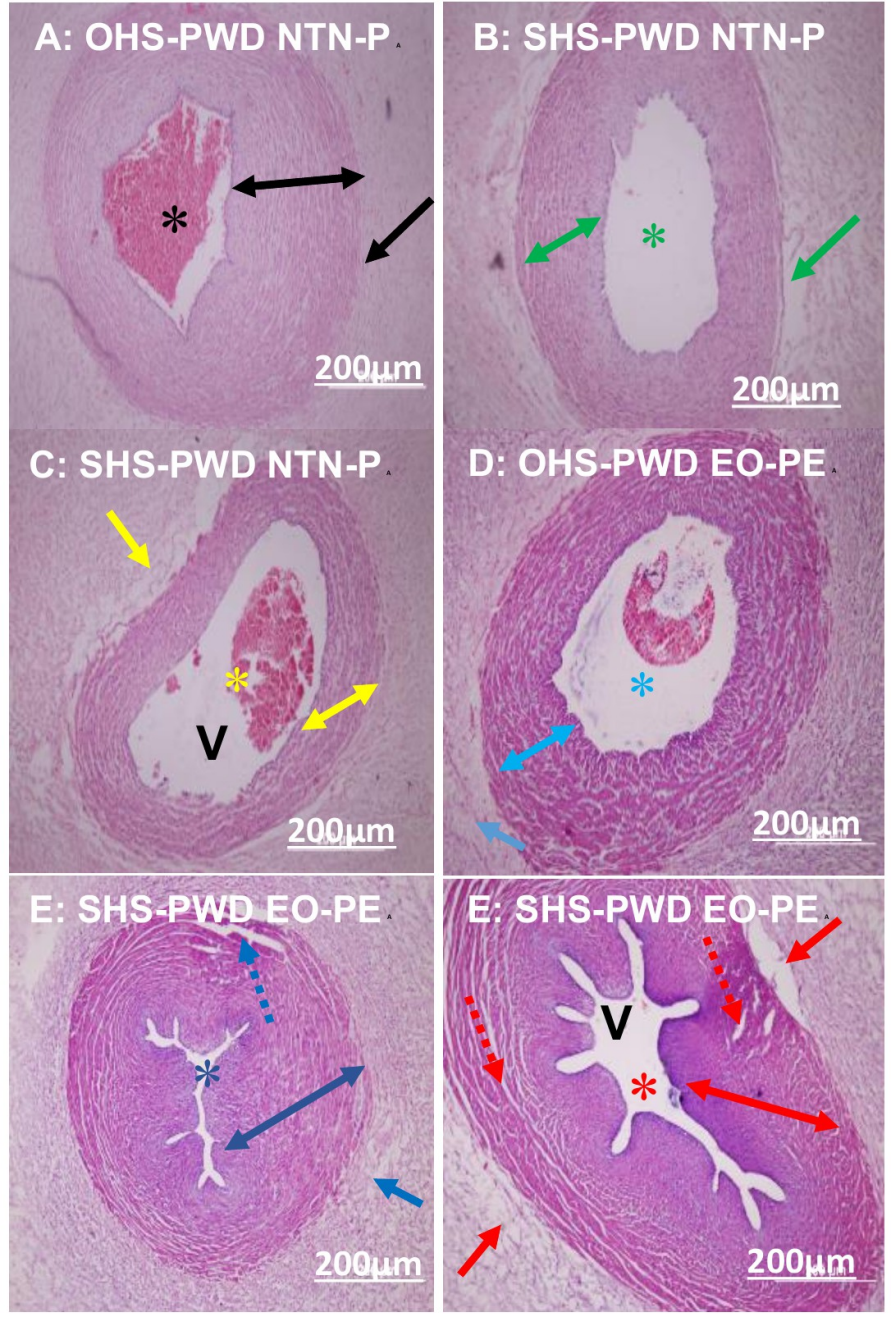
Photomicrographs of umbilical cord artery and vein of SHS and OHS-PWD EO-PE and LO-PE compared to NTN-P placentae. A: intact tunica media of umbilical cord artery (double black arrow) with large lumen (black asterisk) and intact musculostromal architecture (black arrow) of OHS-pregnant women who experienced NTN-P at 40 weeks (magnification x20). B: intact tunica media of umbilical cord artery (double green arrow) with large lumen (green asterisk) and moderate disruption of musculostromal architecture (green arrow) in SHS- pregnant women who experienced NTN-P at 38 weeks (magnification x20). C: intact umbilical cord vein (double arrow) with large lumen (yellow asterisk) and a moderate disruption of musculostromal architecture (yellow arrow) in SHS- pregnant women who experienced NTN-P at 38 weeks (magnification x20). D: intact tunica media of umbilical cord artery with large lumen (sea blue asterisk), few disruptions of musculostromal architecture (sea blue arrow) and a massive vascular wall fibrin deposition (double sea blue arow arrow) in OHS- pregnant women who developed EO-PE at 33 weeks (magnification x20). E: narrowed lumen (deep blue asterisk), few disruptions of the endothelium (deep blue line arrow), vascular wall hypertrophy with vascular wall fibrin depositions (double deep blue arrow) in SHS- pregnant women who developed EO-PE at 32 weeks (magnification x20). F: narrowed lumen umbilical vein (red asterisk), moderate disruption of the endothelium (red line arrow) and musculostromal architecture (red arrow), vascular wall hypertrophy with vascular wall fibrin depositions (double red arrow) in SHS- pregnant women who developed EO-PE at 32 weeks (magnification x20). Scale bar = 200μm.

**Fig 4:**
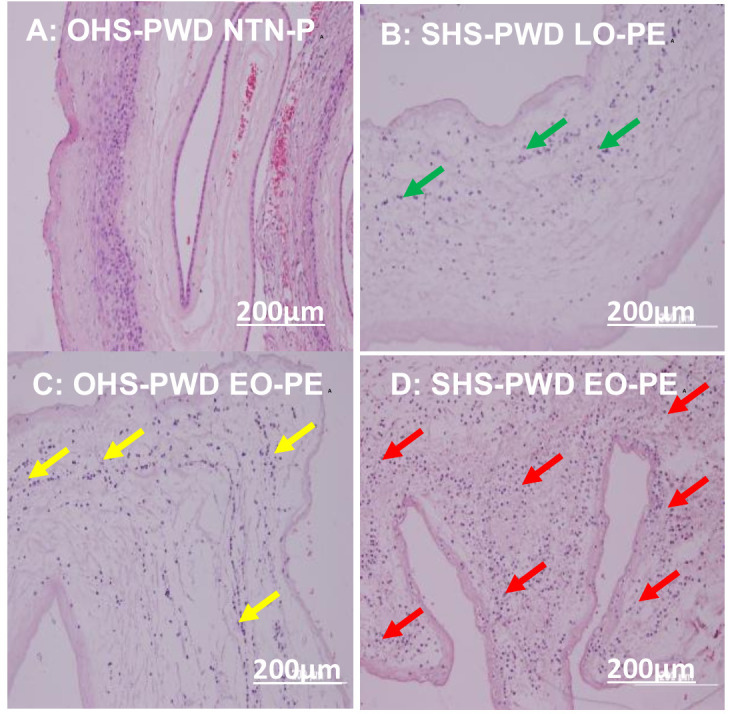
Photomicrographs of chorioamniotic membrane of SHS and OHS-PWD EO-PE and LO-PE compared to NTN-P. A: normal chorioamniotic membrane of OHS- pregnant women who experienced NTN-P at 38 weeks (magnification x20). B: mild acute inflammatory granulocytes infiltrates (chorioamnionitis) (green arrows) in SHS- pregnant women who developed LO-PE at 36 weeks (magnification x20). C: moderate acute inflammatory granulocytes infiltrate (chorioamnionitis) (yellow arrows) in OHS-PWD EO-PE at 33 weeks associated with PPROM (magnification x20). D: severe acute inflammation granulocytes infiltrate (chorioamnionitis) (red arrows) in SHS- pregnant women who developed EO-PE placenta at 32 weeks associated with preterm premature rupture of membrane (PPROM) (magnification x20). Scale bar = 200μm.

The histopathological features in the photomicrographs showed moderate to extensive umbilical cord **(Fig 3)** and chorioamniotic membrane **(Fig 4)** lesions in placentae of SHS rather than OHS pregnant women who developed EO-PE followed by those who developed LO-PE, and a mild to no lesion in NTN-pregnant women. The placental umbilical cord lesions commonly identified were tunica media and vascular wall hypertrophy, vascular wall fibrin formation and distorted musculostromal architecture **(Fig 3)**. Chorioamniotic membrane lesion identified was acute inflammation (chorioamnionitis) **(Fig 4)**.

### Associations and histopathological morphology of the placental umbilical cord and membrane of SHS and OHS pregnant women who developed EO-PE and LO-PE compared to NTN-P

A shown in **Table 5,** a statistically significant higher proportion of umbilical cord artery and vein lesions **(Fig 3)** and chorioamniotic membrane **(Fig 4)** were associated with mothers with SHS rather than OHS pregnant women who developed EO-PE followed by those who developed LO-PE when both were compared to NTN-P (all *p*<0.0001) **(Table 5).**

**Table 5:** Histopathological morphology of placental umbilical cords and membranes among SHS and OHS-PWD EO-PE and LO-PE compared to NTN-P.

Features	SHS-PWD EO-PE (N = 56)	SHS-PWD LO-PE (N = 97)	SHS-PWD NTN-P (N = 95)	*#p-value*	OHS-PWD EO-PE (N = 14)	OHS-PWD LO-PE (N = 30)	OHS-PWD NTN-P (N = 206)	*#p-value*
**Tunica media**				**<0.0001**				**<0.0001**
Normal	45 (80.3)	93 (95.9)	95 (100)		11 (78.6)	30 (100)	206 (100)	
Hypertrophy	11 (19.6)	4 (4.1)	0 (0.0)		3 (21.4)	0 (0.0)	0 (0.0)	
**Vascular wall**				**<0.0001**				**<0.0001**
Intact	46 (82.1)	94 (96.9)	95 (100)		12 (85.7)	30 (100)	206 (100)	
Hypertrophy	10 (17.9)	3(3.1)	0 (0.0)		2 (14.3)	0 (0.0)	0 (0.0)	
**Vascular wall Fibrin**				**<0.0001**				**<0.0001**
Not present	28(50.0)	86(88.7)	94(98.9)		6(42.9)	24(80.0)	206(100)	
Mild	12(21.4)	8(8.2)	1(1.1)		3(21.4)	6(20.0)	0(0.0)	
Moderate	10(17.9)	3(3.1)	0(0.0)		4(28.6)	0(0.0)	0(0.0)	
Extensive	6(10.7)	0(0.0)	0(0.0)		1(7.1)	0(0.0)	0(0.0)	
**Distorted musculostromal architecture**				**<0.0001**				**<0.0001**
Not present	28(50.0)	95(97.9)	94(100)		6(42.9)	28(93.3)	206(100)	
Mild	15(26.8)	4(4.1)	0(0.0)		2 (14.3)	2(6.7)	0(0.0)	
Moderate	8(14.3)	1(1.0)	0(0.0)		6(42.9)	0(0.0)	0(0.0)	
Severe	5(8.9)	0(0.0)	0(0.0)		0(0)	0(0.0)	0(0.0)	
**Membrane chorioamnionitis**				**<0.0001**				**<0.0001**
Not present	26(46.4)	87(89.7)	95(100)		6(42.9)	26(86.7)	206(100)	
Mild	8(14.3)	8(8.2)	0(0.0)		1(7.1)	4(13.3)	0(0.0)	
Moderate	14(25.0)	2(2.1)	0(0.0)		5(35.7)	0(0.0)	0(0.0)	
Extensive	8(14.3)	0(0.0)	0(0.0)		2 (14.3)	0(0.0)	0(0.0)	

Values are presented as frequency (percentages). #p-value indicates p-values within group comparison. SHS: suboptimal health status; OHS: optimal health status; PWD: pregnant women who developed; EO-PE: early-onset PE; LO-PE: late-onset PE; NTN-PW: normotensive pregnancy women

P-values < 0.05 in bold indicates statistical significance.

Association was tested using Chi-square test for trend.

### Immunohistochemical expression and semi-quantitative evaluation of placental pro-AGMs (PIGF and VEGF-A), anti-AGMs (Eng and Flt-1) and oxidative DNA marker (8-OHdG) in placentae of SHS and OHS-P pregnant women who developed EO-PE and LO-PE compared to NTN-P

**Fig 5:**
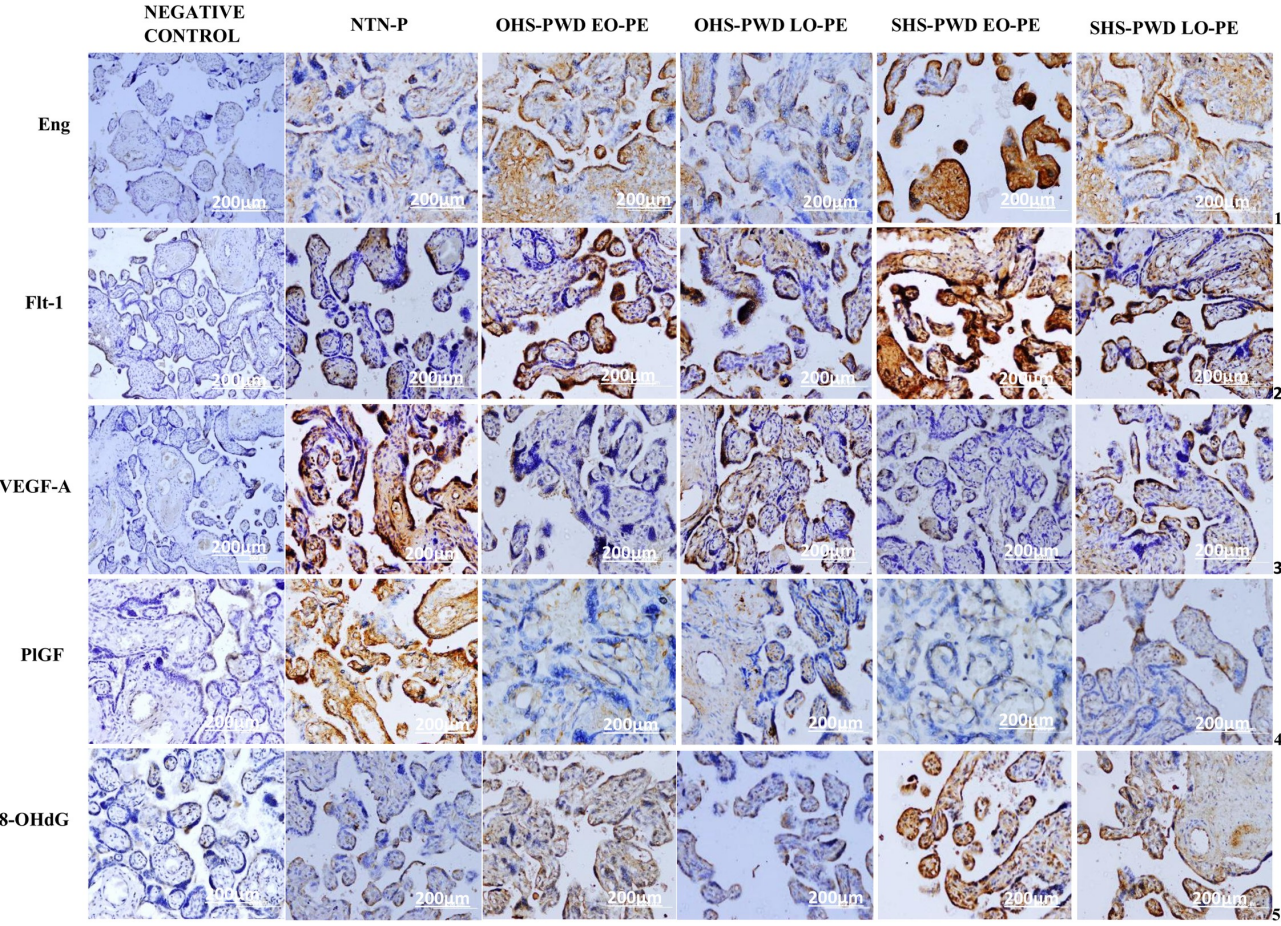
Photomicrographs of the differential expression of pro-AGMs (PIGF and VEGF-A), anti-AGMs (Eng and Flt-1), and oxidative DNA marker (8-OHdG) in placentae from SHS and OHS-PWD EO-PE and LO-PE compared to NTN-P. Negative control showing negative or no IHC staining (column 1). Higher staining intensity/diffuse positivity (high brown staining) of expression of Eng (row 1), Flt-1 (row 2) in cytotrophoblast, syncytiotrophoblast, mesenchymal cells of the villous stroma and syncytial knots of SHS- pregnant women who developed EO-PE at 32 weeks as compared to OHS- pregnant women who developed EO-PE at 32 weeks gestation. Partial positivity (moderate brown staining) expression in SHS- pregnant women who developed LO-PE at 37 weeks, focal positivity (low brown staining) expression in OHS- pregnant women who developed LO-PE at 37 weeks and negative expression in NTN-pregnant women at 38 weeks. Higher staining intensity/diffuse positivity (high brown staining) of expression of VEGF (row 3) and PlGF (row 4) in endothelial cells of foetal capillaries and syncytiotrophoblast of NTN-pregnant women at 38 weeks. Focal positivity (low brown staining) expression in SHS and OHS- pregnant women who developed LO-PE at 37 weeks and negative expression in SHS and OHS- pregnant women who developed EO-PE at 32 weeks. Higher staining intensity/diffuse positivity (high brown staining) of expression of 8-OHdG (row 5) in cytotrophoblast cell nuclei and syncytiotrophoblast of SHS- pregnant women who developed EO-PE and OHS- pregnant women who developed EO-PE at 32 weeks gestation. Partial positivity (moderate brown staining) expression in SHS- pregnant women who developed LO-PE at 37 weeks, focal positivity (low brown staining)/negative expression in OHS- pregnant women who developed LO-PE at 37 weeks and NTN-pregnant women at 38 weeks (magnification x20). Scale bar = 200μm.

As shown in **Fig 5**, the photomicrographs showed expression of antibodies, Eng (Fig 5, row 1) and Flt-1 **(Fig 5** row 2**)** in syncytiotrophoblast, cytotrophoblast and mesenchymal cells of the villous stroma, whereas VEGF-A (**Fig 5**, row 3) and PlGF (Fig 5, row 4) were expressed in endothelial cells of foetal capillaries and syncytiotrophoblast. The oxidative DNA damage marker, 8-OHdG (**Fig 5**, row 5), was expressed in cytotrophoblast nuclei and syncytiotrophoblast. Negative IHC controls for angiogenic growth mediators and the DNA damage marker antibodies show no staining (**Fig 5** column 1) **(Fig 5).**

### Quantitative analysis of expression levels of angiogenic growth mediator and DNA damage marker antibodies

**Fig 6:**
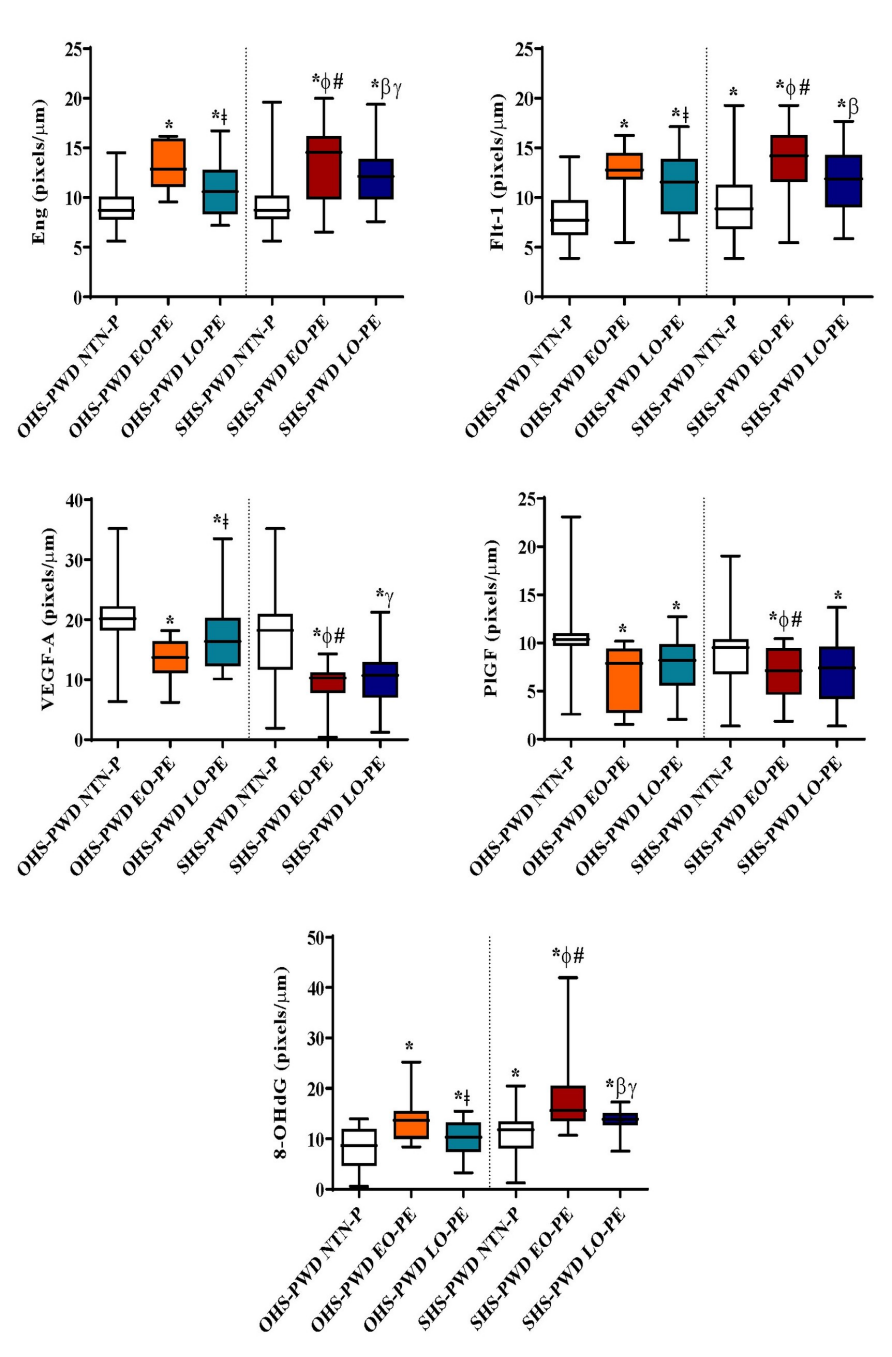
Image J quantitative analysis of expression level in pixels/μm of Eng, Flt-1, PIGF, VEGF-A and 8-OHdG in placentae from SHS and OHS-PWD EO-PE and LO-PE compared to NTN-P. * indicates significance compared to OHS-PWD NTN-P. ǂ indicates significance between OHS-PWD LO-PE and OHS-PWD EO-PE. ɸ indicates significance between SHS-PWD EO-PE and OHS-PWD EO-PE. # indicates significance between SHS-PWD EO-PE and OHS-PWD LO-PE. β indicates significance between SHS-PWD LO-PE and SHS-PWD EO-PE. γ indicates significance between SHS-PWD LO-PE and OHS-PWD LO-PE.

When the software Image J was used to quantify the intensity of expression as shown in **Fig 6**, a significantly higher level of expression of antibodies Eng, Flt-1 and 8-OHdG and low expression of PIGF and VEGF were observed in the placentae of SHS-pregnant women who developed PE rather than OHS-pregnant women who developed PE, particularly EO-PE>LO-PE when compared to NTN-pregnant women (all *p*<0.0001) **(Fig 6).**

## Discussion

The present study examined the histopathological features, serum and immunohistochemical expression of pro-and anti-AGMs in addition to an oxidative DNA marker in placentae from suboptimal health status (SHS) and optimal health status (OHS)-pregnant women, who later developed PE, specifically EO-PE and LO-PE and compared it to placentae from NTN-PW in a Ghanaian population. This study is the first to incorporate SHS concepts in histopathological and immunohistochemical differential diagnoses among PE and normotensive pregnancies. At baseline, we observed a higher imbalance in AGMs and OS biomarkers among SHS normotensive pregnant women. This imbalance corresponded with abnormal histopathological and immunohistochemical expression of OS and AGMs among women who developed PE.

In the present study, the new cases of PE found among SHS, and OHS mothers were 30.7% and 8.8%, respectively. An incidence rate of PE was 9.0% among Ghanaian pregnant women visiting the Komfo Anokye Teaching Hospital [23, 24], and is similar to the incidence of 8.8% recorded among OHS in the present study. Conversely, the unexpectedly high incidence rate of PE among SHS pregnant women is the first to be reported, and the probable explanation is not self-understood. The concept of SHS as a risk indicator of PE development is new. Previous studies among a general adult population have reported an association of SHS with endothelial dysfunction [17], cardiovascular disease [18] and adverse pregnancy outcomes [19]. A recent study by Anto et al. [19] reported that SHS pregnant women were at a high-risk incidence of PE compared to OHS pregnant women. This observation indicates that diagnosing normotensive pregnancies based on blood pressure and proteinuria alone is insufficient. Some normotensive women have subclinical or poor health without visible symptoms, also known as suboptimal health status. Hence, the concept of SHS, when used in addition to standard criteria for antenatal care assessments, may help identify pregnant women at high risk of developing PE.

In the present study, there was an imbalance in the biomarkers of OS as indicated by a significantly reduced plasma TAC and correspondingly increased levels of serum 8-epiPGF2α, 8-OHdG, and urinary 8-OHdG among SHS and OHS pregnant women who developed PE, particularly EO-PE rather than LO-PE compared to NTN_pregnancy **(Table 1)**. The degree of imbalance was more in favour of the SHS groups rather than the OHS group. In a cross-sectional study, Anto et al. [20] found a significant imbalance in OS biomarkers among SHS pregnant women compared to OHS pregnant women, which is in consonance with the present finding [20]. This finding indicates high endogenous peroxidation, oxidative DNA damage and a compromised antioxidant system, which is common in SHS pregnant women who develop PE compared to OHS pregnant women. In PE women, increased OS may originate from placental hypoxia and ischaemia, high demands of the foetus and the pregnancy that further induce systemic reactive oxygen species (ROS) production and cause damage to lipids, DNA and proteins. Hence, SHS precede the clinical manifestation of PE.

Another significant novel finding of the present study was an imbalance in AGMs as indicated by a significantly reduced PIGF and VEGF-A. It correspondingly showed increased levels of sEng, sFlt-1, sFlt-1/PlGF ratio, and sEng/PlGF ratio among SHS and OHS pregnant women. At the time of delivery, they developed PE, particularly in EO-PE women followed by LO-PE, when they were both compared to NTN pregnancies (Table 2). Previous case-control studies have reported imbalances in AGMs [25–27] among PE compared to controls. Several longitudinal studies [28, 29] have reported that imbalances in AGMs can occur at early gestation before the clinical manifestation of PE. Our study, which showed a massive imbalance in the levels of pro-AGMs and anti-AGMs in SHS who developed PE more than OHS pregnant women when compared to NTN pregnancies, may be explained by the significant imbalance at baseline. This observation suggests that SHS mothers are at high risk of defective angiogenesis and vasculogenesis. Thus the concept of SHS may be essential and valuable in pregnancy, especially among mothers from resourced limited countries. The mechanism may operate via shallow extravillous trophoblast invasion and subsequent poor maternal artery remodelling resulting in placental under perfusion and hypoxia [30].

In the present study, the gestational age at delivery, birthweight, placental weight and placental thickness were statistically significantly reduced, whereas the SBP, DBP, foetoplacental weight (FPW) ratio and maternal age were significantly increased among SHS PE subtypes more than the OHS PE subtypes, specifically in EO-PE rather than LO-PE when both were compared to NTN-pregnant women. A prospective cohort study conducted by Krielessi et al. [31] among pregnant women in Greece and a case-control study conducted by Nahar et al. [32] in Bangladesh reported high rates of preterm delivery, low birthweight and placental weight, and reduced placental thickness in preeclamptic births as opposed to NTN-P. The FPW ratio is a measure of placental nutritional efficacy; hence, an increased ratio is suggestive of placental insufficiency and severity of the PE condition [31, 33–36]. However, the FPW may vary between populations due to environmental, epigenetic and metabolic factors [36]. Unlike normotensive pregnancies, increased maternal age is mainly associated with adverse pregnancy outcomes like PE [27,37,38]. The findings here clearly explain the clinical manifestations associated with PE. Also, high rates of adverse events such as stillbirth, PPROM and low APGAR score were more related to the SHS_PE group followed by the OHS group, suggesting that pregnant women with SHS at early gestation are the high-risk population for PE development.

Another finding was the significant difference in gross placental lesions between the study groups who developed PE compared to NTN pregnant women. The most common lesions identified on gross placental examination were placental infarctions, calcifications, distorted architecture of villus cotyledons and membrane rupture sites, and all these lesions were significant, with higher proportions associated with placentae from SHS_PE subtypes than OHS_PE subtypes and most specifically in EO-PE rather than LO-PE compared to NTN-pregnant women. These observations are indicative of the severity of the PE condition; particularly, EO-PE pregnancy is associated with pregnancy complications that contribute to abnormal placental lesions [5]. Placental infarct occurs due to uteroplacental ischaemia and may be associated with IUGR and foetal demise with or without PE [39, 40].

Based on the gross placental findings, a further microscopic examination was performed to understand the histopathological morphology of the placental lesions. The present study observed a significantly increased number and proportion of intravillous/perivillous fibrin, fibrinoid necrosis, syncytial knot formations, infarction and chorangiosis in SHS_PE placentae compared to the OHS_PE placentae, and this was more extensive, particularly in EO-PE versus LO-PE placentae, as compared to those from NTN-PW. Perivillous fibrin deposits and fibrinoid necrosis, although rare, are associated with fetal growth restriction (FGR) and foetal demise [41]. This explanation supports the results of the present study, which observed that the EO-PE showed a higher incidence of FGR and foetal demise. The extensive fibrin deposition could be explained by hypoxia-associated apoptosis or necrosis of syncytiotrophoblast originating from incomplete placental angiogenesis [31]. Villous fibrinoid necrosis may be due to placental ischaemia in response to placental insufficiency. In this study, findings of increased syncytial knots in EO-PE are consistent with those reported by Weel et al.[5] and Kovo et al.[42]. Syncytial knots are commonly seen in mature placental villi; however, their formation in placentae from EO-PE births indicates the severity of the condition due to trophoblastic ischaemia and hypoxia or FGR [43, 44]. Besides, an increased proportion of placental infarction observed in the present study could be due to hypoxia/ischaemia originating from the reduced exchange of blood nutrients within the intervillous space [45, 46].

In the present study, the placentae from PE groups had significantly reduced umbilical cord lengths, consistent with the finding of a population-based study by Linde et al.[47]. Short umbilical cord length is usually associated with foetal distress and FGR and neonatal asphyxia [47]. In addition, an increased proportion of tunica media hypertrophy, vascular wall hypertrophy and vascular wall fibrin deposition were commonly observed among SHS_PE subtypes, more than OHS_PE subtypes, particularly in EO-PE. A related observation was made in LO-PE when both were compared to NTN-pregnant women. These findings are similar to those reported by some case-control studies [48, 49]; however, these studies did not examine these lesions in SHS and OHS-embodied PE. The observed lesions may also be due to increased vascular resistance originating from incomplete angiogenesis and placental foetal-hypoperfusion [50].

Chorioamnionitis is a placental membrane lesion linked to preterm births, infections during labour, spontaneous labour and premature rupture of membrane (PROM) [51]. In the present study, a significantly increased proportion of acute inflammation/chorioamnionitis was observed in chorioamniotic placental membranes from SHS_PE subtype more than OHS_PE subtype, particularly EO-PE rather than LO-PE when both were compared to NTN-pregnant women. It is probable that the increased proportion of PROM and preterm births, which were common among the PE groups in the present study, may have contributed to chorioamnionitis.

An imbalance in placental pro-and anti-AGMs levels is associated with EO-PE compared to LO-PE [5, 52]. The present study found PlGF, VEGF, Flt-1 and Eng expression in the syncytiotrophoblast **(Fig 5)**. Besides, VEGF-A and Flt-1 were also expressed in the cytoplasm of mesenchymal cells and the endothelium of foetal capillaries. Higher positivity of Flt-1 and Eng expression was found in the SHS PE subtype compared to the OHS_PE subtype, particularly, EO-PE followed by LO-PE when both were compared to NTN pregnant women. Conversely, increased positivity of VEGF-A and PlGF were found in NTN pregnant women with focal or negative staining in EO-PE placentae. The present study finding agrees with a case-control study by Weel et al. [39], who found a higher expression of Flt-1 and low expression of PIGF and VEGF are associated more with EO-PE than LO-PE. Gu et al. [9] found an increased expression of Eng and Flt-1 and reduced immunopositivity of PIGF in PE placentae compared to NTN-placentae. The results presented here suggest the occurrence of abnormalities in factors responsible for regulating placental angiogenesis and vasculogenesis are commonly associated with SHS-embodied EO-PE. The placental abnormalities found on IHC may be explained by placental ischaemia and hypoxia due to placental insufficiency [5]. Placental ischaemia stimulates the increased release of hypoxia-inducible factor, a ROS-sensitive transcription factor that can induce membranous Flt-1 and release sFlt-1 from villous cytotrophoblast cells, which are connected to the intervillous spaces [5, 53]. The higher positive immunoexpression of Flt-1 protein may be responsible for the reduced expression of PIGF and VEGF-A [54]. Also, the increased immunopositive expression of Eng in EO-PE placentae further suggest endothelial cell dysfunction [9].

In another finding of the present study, the 8-OHdG was highly expressed in placental cytotrophoblast nuclei and syncytiotrophoblast, of which a significantly higher immunoexpression was detected in placentae from the SHS_PE groups, more than OHS PE group, mainly the EO-PE followed by LO-PE when both were compared to NTN-pregnant women **(Fig 5)**. This finding supports the reports that the extent of oxidative DNA damage depends on the severity of the PE and IUGR [7, 12]. The present study found a significantly higher proportion of FGR complicated by EO-PE, which may further explain the increased intensity of 8-OHdG immunopositivity in the PE placentae. The placental oxidative DNA damage may be due to ischaemia-reperfusion injury associated with severe PE [13, 55, 56].

The present study quantified the immunopositivity using software Image J and found a significantly higher expression of Flt-1, Eng and 8-OHdG immunopositivity but a reduced expression of PIGF and VEGF-A immunostaining intensity in the placentae from the SHS_PE groups more than OHS_PE group, particularly the EO-PE followed by LO-PE when both were compared to NTN pregnant women **(Fig 6).** This observation allows differentiation of the PE subtypes. The present study hypothesises that the imbalance in pro-and anti-AGMs and increased 8-OHdG caused by placental hypoxia contributes to increased placental lesions; thus, underscoring their use as potential prognostic markers for PE. From a prophylactic and therapeutic standpoint, a combination of pro-angiogenic molecules and antioxidant supplementation for mothers suspected as susceptible to developing PE would be appropriate to maintain the placental physiology while sustaining the growing foetus and the wellbeing of the pregnant mother.

Despite these findings, there were some limitations. One of them is that the present study did not evaluate these antibodies in placental homogenate to determine the placental levels by ELISA. Also, the present study did not perform western blots to standardise the quantification of AGMs and 8-OHdG. Nevertheless, the present study is the first to investigate concurrently the histopathological presentation of the placental lesions and their immunohistochemical expression patterns of pro-and anti-AGMs and 8-OhdG from SHS and OHS-pregnant women who developed EO-PE and LO-PE compared to NTN pregnant women, in a nested case-control study among a Ghanaian population.

## Conclusion

Early imbalance of serum levels of oxidative stress (OS) biomarkers and angiogenic growth mediators (AGMs) was observed among suboptimal health status (SHS) normotensive pregnant women, and this imbalance corresponded with abnormal histomorphological features and immunohistochemical expression of OS and AGMs among women who developed PE, suggesting the potential involvement of SHS in the pathophysiology of PE. There are more abnormal placental lesions, a reduced expression of pro-AGMs (PIGF and VEGF-A) and a correspondingly increased expression of anti-AGMs (Flt-1 and Eng) and the oxidative DNA damage marker (8-OHdG) in the placentae from SHS rather than OHS pregnant women, who developed EO-PE compared to those who developed LO-PE. Unlike OHS pregnant women, SHS pregnant women are the high-risk PE population, thus allowing differential placental evaluation of PE conditions. Integration of SHS evaluation as an additional health assessment criterion for pregnant women from low-resourced countries would prompt clinicians the need for early prophylaxis and subclinical symptoms, which may result in PE.

From a prophylactic point of view, we hypothesise that supplementation with antioxidants in combination with pro-angiogenic molecules to SHS normotensive pregnant women in early pregnancy would maintain the placental physiology and improve the wellbeing of both the pregnant mother and the growing foetus while preventing the development of PE.
